# Explainable Structured Pruning of BERT via Mutual Information

**DOI:** 10.3390/e27121224

**Published:** 2025-12-02

**Authors:** Hanjuan Huang, Hao-Jia Song, Qiling Zhao

**Affiliations:** 1 College of Mechanical and Electrical Engineering, Wuyi University, Wuyishan 354300, China; huanghanjuan@wuyiu.edu.cn; 2 Department of Computer Science and Information Engineering National, Taiwan University of Science and Technology, Taipei 10607, Taiwan; hhh5911@gmail.com; 3 Fujian Key Laboratory of Big Data Application and Intellectualization for Tea Industry, Wuyi University, Wuyishan 354300, China

**Keywords:** BERT compression, structured pruning, mutual information, explainable

## Abstract

Bidirectional Encoder Representations from Transformers (BERT) excels in natural language processing (NLP) but is costly on edge devices. We introduce an unsupervised, retraining-free structured pruning scheme for BERT, guided by mutual information (MI). Leveraging Rényi α-order entropy, we design a representation-aware MI estimator and a principled kernel-bandwidth selection, producing stable, sample-efficient neuron-level pruning signals. This method removes redundant units while preserving representational capacity, reduces memory and latency, and deploys readily on commodity hardware. Explainable-AI visualizations clarify how compression reshapes intermediate features and predictions. Across benchmarks, the compressed models maintain minimal accuracy loss, outperform or match strong unsupervised baselines, and remain competitive with supervised alternatives.

## 1. Introduction

Bidirectional Encoder Representations from Transformers (BERT)-style large language models deliver outstanding results on diverse natural language processing (NLP) tasks; however, their very large parameter scales and computation costs translate into tight requirements for storage, latency, and power, hindering efficient deployment on resource-limited end/edge devices. Thus, compressing the models without sacrificing the performance has emerged as a key challenge of broad interest to researchers and practitioners.

The most recent progress in BERT compression includes direct network pruning, knowledge distillation, quantization, and low-rank factorization. The different methods enforce the compression of different network parts. Direct network pruning [1,2,3,4,5] aims to remove redundant or unimportant components (neurons or weights) to produce a smaller network. Knowledge distillation [6,7,8] is focused on finding a small student model that learns from a large teacher model. Mimicking the prediction ability of the large model in a small model, we achieve the goal of model downsizing. Quantization [9,10,11] provides another approach to save space by utilizing integers or discrete numbers to substitute for floating-point numbers in networks. The computation time may also be reduced by this design. The last approach, called low-rank factorization [12,13], replaces the large-weight matrix with a small one.

Focusing on the family of network pruning techniques, we consider two types of methods: structured and unstructured. The overall goal is to remove redundant components from the models. First, structured pruning simplifies a BERT by removing entire structural components, such as neurons, channels, or layers while retaining the network structures [14,15]. On the other hand, unstructured pruning [5,16] aims to prune redundant neurons or links. The approach of deleting individual parameters may suffer from irregular sparse structure problems. Comparing the two, structured pruning can be deployed to various edge devices directly, while unstructured pruning must be accompanied by extra software or hardware treatment to complete the task [15].

There are a few items to consider in designing an appropriate compression method for the BERT model. First, quite a few BERT models were trained mainly on unlabeled data. Some of these pretrained LLMs can be used by third-party developers for various downstream tasks. Therefore, we do not know and we cannot assume that any label or contextual information is provided when building the LLMs. That is to say, we do not know the real aim when building the models or applying any compression to the models [17]. Hence, we should only assume an unsupervised type of compression applied to LLMs. Second, most pruning methods need retraining to maintain the model performance. This retraining procedure inevitably consumes resources [17] and should be avoided if possible. In BERT models, the feed-forward network (FFN) is the major part of the models in terms of size, and it requires the most computation [18]. Therefore, compressing this part of a network can save space and time complexity. All the above points motivate us to search for an unsupervised BERT compression method that needs no further retraining after the compression is performed. Moreover, we prefer one pruning method that can be applied to FFN in particular: structured.

Among structured pruning paradigms, mutual information (MI)-based approaches quantify the informational relevance between network units and inputs/labels, thereby providing a principled criterion for ‘what to retain’ versus ‘what to prune.’ However, high-dimensional MI estimation in deep networks remains challenging: the bias–variance trade-off is difficult to balance, and the sample efficiency and stability are often inadequate in practice. To address these issues, building on the prior work of Wickstrøm et al. [19], we propose an MI estimation method tailored to the feature distributions of deep neural networks. Specifically, we estimate the MI between hidden neurons using Rényi’s α-order entropy estimator and show that the kernel bandwidth parameter σ is pivotal for the estimation accuracy. We further introduce a principled bandwidth selection strategy that enhances the fidelity, stability, and sample efficiency of the estimator, thereby yielding more reliable pruning signals. Using these improved estimates, we accurately remove redundant neurons to obtain compact, yet effective, models, preserving the original performance at substantially higher compression ratios and improving the robustness and practical operability of MI-guided structured pruning.

Although numerous pruning methods achieve strong quantitative results, their interpretability remains limited [20]. The black-box nature of deep neural networks raises legitimate concerns regarding the reliability of compressed models, particularly when substantial parameter reductions are required while preserving near-original task performance. Rajapaksha et al. [21] employed explainable AI (XAI) techniques to elucidate model decisions and predictions, thereby promoting transparency. Inspired by this line of work, we likewise incorporate XAI to enhance the transparency and trustworthiness of our approach. Building on our previous study on the interpretability of pruning methods [22], which primarily focused on explaining the pruning algorithms themselves, the present work shifts attention to elucidating why the compressed models can maintain performance despite extensive parameter reduction.

In summary, we propose a mutual information-based structured pruning method that primarily targets the FFN layers of BERT. We further develop an MI estimation technique tailored to deep networks and integrate visualization analyses to intuitively explain the effects of compression.

We summarize the proposed work and its contributions as follows:

- We propose a novel MI-based structured scheme targeting BERT’s FFN layers, achieving high compression with minimal accuracy loss for on-device deployment.
- The proposed method is considered an unsupervised approach, which needs no label information to decide the pruning strategy. That provides a lower burden when moving to large-scale models, which may suffer from the labeled data-hungry problem.
- A mutual information estimation method tailored to deep representations is proposed, featuring a novel kernel bandwidth estimator to compute MI between hidden nodes.
- We construct visualizations of the compression process to intuitively reveal changes in the representations and predictive behavior before and after pruning, thereby enhancing understanding and trust in the method’s effectiveness.
- The method is superior to other unsupervised pruning methods. It also shows some competitiveness when compared to some of the supervised approaches.

The rest of the paper is organized as follows. We elaborate on the background knowledge related to model pruning and BERT in Section 2, which is followed by a detailed explanation of the proposed model pruning methodology and the estimation techniques in Section 3. To evaluate the proposed method, we present the results and discussion in Section 4, and, in Section 6, we conclude this work. This article is an extended version of our preprint available at arXiv:2406.00030 [23].

## 2. Related Work

In this section, we review the work related to deep model pruning. This review includes various pruning methods and introduces mutual information (MI) estimation, which is the metric guiding our proposed procedure.

### 2.1. Structured Pruning

We primarily review the research on structured pruning for large-scale models.

Voita et al. [24] proposed a pruning method based on stochastic gates and a differentiable relaxation of the $L_{0}$ penalty, capable of pruning a majority of attention heads without seriously affecting the model’s performance. Liu et al. [25] proposed a structured pruning method for efficient BERT inference (EBERT), which can dynamically prune unimportant heads in multi-head self-attention (MHA) and unimportant channels in FFN with the help of the predictor branch. Labeled data are necessary for these operations. Kwon et al. [26] proposed a three-stage pruning framework, which used a Fisher-based mask search algorithm (labeled data are needed) to decide which heads/filters to prune, then rearranged the pruned heads/filters, and finally tuned the mask variables to recover the output signal for each layer. Yang et al. [27] proposed a model pruning toolkit called TextPruner for pretrained language models. The toolkit includes two pruning methods: the supervised method used the training loss to measure the importance score of neurons; the self-supervised method used the Kullback–Leibler divergence to measure the importance score of neurons. Park et al. [28] proposed a structured pruning algorithm, named Kprune (knowledge-preserving), which focused on preserving the useful knowledge of the pretrained model to minimize pruning errors through an iterative pruning process that consisted of knowledge measurement, knowledge-preserving mask search, and knowledge-preserving weight-tuning. Ma et al. [4] introduced an LLM pruning approach named LLM-Pruner. This method employed structural pruning, selectively eliminating non-essential coupled structures guided by gradient information. The aim is to preserve the LLM’s functionality to the fullest extent possible. Nova et al. [17] proposed a gradient-free structured pruning framework to integrate two ranking techniques: representative ranking and data-driven ranking, without the help of labeled data. While these studies effectively compress large models, several rely on labeled data (e.g., [4,24,25,26,27,28]), whereas others require post-pruning retraining/fine-tuning (e.g., [4,25,29]).

### 2.2. Mutual Information Estimation

MI estimation on deep learning networks is difficult, if not intractable, due to the large scale of the network structures and the data size. The classical binning-based estimator [30] considered quantizing neurons’ output to estimate the corresponding probability distribution, which leads to at least three problems: (1) there must be an appropriate decision on the bin size to ensure the estimation precision [31,32]; (2) probability distribution estimation needs a large number of samples [32]; (3) it is difficult to compute certain activation functions such as ReLU. Some other issues include the systematic errors [33] that may occur in the computation procedure. We can utilize some hyperbolic functions (e.g., tanh) to deal with this last issue. Kraskov et al. [34] proposed a *k*NN distance-based MI estimation called KSG, to deal with a wide range of activation functions. However, this relies on a wise decision as to the number of neighbors. To deal with other issues, Belghazi et al. [35] proposed the Mutual Information Neural Estimator (MINE), adopting a different network, using gradient descent to realize MI estimation given high-dimensional random variables. Through their framework, both the dimensionality of neurons and the number of samples can be extended linearly for better estimation precision. However, the method is sensitive to the choice of network, and the convergence speed is slow for such a network. Wickstrøm et al. [19] improved on the results of Giraldo et al. [36] and Yu et al. [37] and proposed a novel matrix- or tensor-based estimation called the Rényi α-order entropy estimator, which can estimate MI for high-dimensional multivariate data without estimating the probability of the random variables involved in the MI computation.

The key to the Rényi α-order entropy estimator is the estimation of the kernel width parameter. One can choose between supervised and unsupervised learning approaches. In supervised learning, an optimal criterion [19] was used, while Scott’s rule [38] was considered for the unsupervised learning case. In brief, they aligned the label kernel matrix and a kernel matrix from a pre-specified layer to approximate the kernel width parameter. Using Scott’s rule [38], they estimated the kernel width parameter by examining the data size and the dimensionality of the focused hidden layer. The current approach to applying Scott’s rule is the estimation of the whole hidden layer [32].

Table 1 summarizes the core principles and key limitations of various MI estimation techniques, particularly concerning their application in deep network analysis.

## 3. Proposed Method

In this section, we detail the proposed method. Given a pretrained BERT, we perform a fine-tuning procedure that is designed for a specific task and then apply the proposed pruning to the fine-tuned model to obtain its compressed version. The compressed model is assumed to have similar behavior to the original model.

Before we elaborate the details of the proposed method, we introduce the notations used in this work.

### 3.1. Preliminaries

#### 3.1.1. Notations

In the *ℓ*-th transformer encoder, we have $K_{\ell }$ neurons in the fully connected layer of FFN, which are denoted by $Z_{1},Z_{2},\ldots ,Z_{K_{\ell }}$, and we use the random variables $Z_{1},Z_{2},\ldots ,Z_{K_{\ell }}$ to describe the value of those neurons (features) in the FC layer. That is, Z refers to a neuron, and *Z* is the random variable to describe the value on neuron Z. $I(Z_{k};Z_{\ell })$ denotes the MI between the random variables.

#### 3.1.2. Information-Theoretic Basis

To ensure methodological transparency, we first introduce the mathematical formalisms underpinning our neuron evaluation strategy. Our approach relies fundamentally on MI estimation, which is closely related to entropy:

- MI: MI I(X;Y) quantifies the dependence between two random variables *X* and *Y*. It is formally defined using the standard Shannon entropy H(·) as
  I(X;Y)=H(X)+H(Y)−H(X,Y).
  In the context of model pruning, the goal is to assess the shared information between a neuron’s output (*X*) and the model’s output or target labels (*Y*).
- Rényi α-Order Entropy: To facilitate a robust non-parametric estimation of MI, we leverage the Rényi α-order entropy ($H_{\alpha }\left(X\right)$). Unlike Shannon entropy, Rényi entropy is particularly useful when probability density functions are difficult to estimate directly. It is defined as
  $$H_{\alpha }\left(X\right)=\frac{1}{1-\alpha }\log\left(\sum_{x}p{\left(x\right)}^{\alpha }\right),$$
  where p(x) is the probability mass function for a discrete variable *X*, and α>0 with α≠1.

### 3.2. Framework

**Figure 1:**
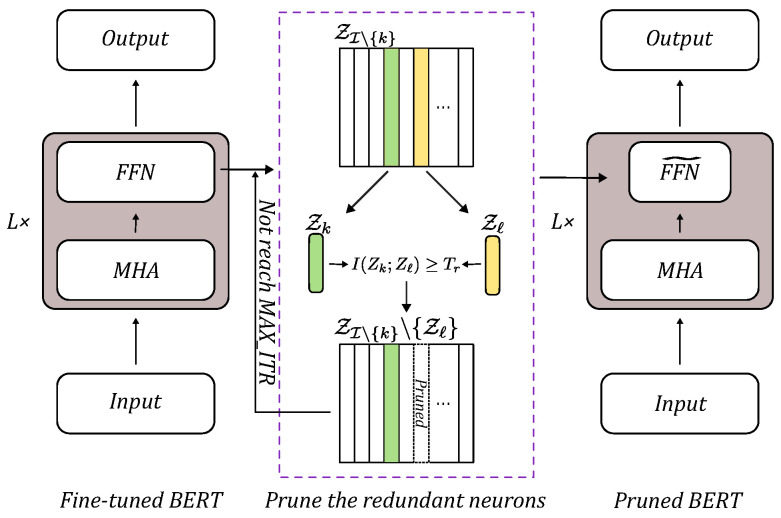
The flowchart for BERT.

The overall architecture is presented in Figure 1. We adopt a standard Transformer encoder, in which each block comprises a multi-head self-attention (MHA) module followed by a feed-forward network (FFN); each FFN contains two linear transformations separated by a GeLU activation. Starting from a pretrained BERT with *L* transformer blocks, we fine-tune the model on the target task and then apply the proposed pruning to the FFN’s fully connected (FC) layer. Concretely, within the target FC layer, we first shortlist the top-*k* redundant neurons. At each iteration, we uniformly sample two candidates *k* and *ℓ* from this shortlist and compute the mutual information between their activations, $I(Z_{k};Z_{\ell })$. If $I\left(Z_{k};Z_{\ell }\right)<T_{r}$, one member of the pair is removed; otherwise, both are retained. The iterations continue until the pruning budget is satisfied, yielding a compact model intended to preserve the behavior of the fine-tuned baseline.

### 3.3. Redundancy as a Feature Selection Criterion

We adopt mutual information to measure the relationship between features. Based on the result, we prune features with a certain level of redundancy. In the fully connected layer of FFN, we randomly select two features represented by their corresponding random variables $Z_{k}$ and $Z_{\ell }$, and compute their mutual information as $I(Z_{k};Z_{\ell })$. If the values of $I(Z_{k};Z_{\ell })$ are large enough to show a certain degree of information overlap, we choose one to delete from the feature set.

The whole procedure of the pruning strategy is shown in Algorithm 1.
**Algorithm 1** The algorithm of an alternative strategy to select a subset of features that has low mutual information between pairwise features**Require:**
     $K_{r}$: The no. of remaining features after the alternative pruning strategy     $T_{r}$: Maximum allowed feature overlapping     MAX_ITR: The maximum number of iterations **Ensure:**     $Z_{I\setminus \{k\}}$: The resulting feature set after the pruning strategy 1:$Z_{I\setminus \{k\}}\leftarrow Z$2:$K_{r}=K$3:**for** i=1,…,MAX_ITR **do**4:    Randomly choose two features $Z_{k}$ and $Z_{\ell }$ from $Z_{I\setminus \{k\}}$ with their content described by $Z_{k}$ and $Z_{\ell }$5:    Calculate the mutual information $I(Z_{k};Z_{\ell })$6:    **if** $I\left(Z_{k};Z_{\ell }\right)\geq T_{r}$ **then**7:        $Z_{I\setminus \{k\}}\leftarrow Z_{I\setminus \{k\}}\setminus \left\{Z_{\ell }\right\}$     // Turn off one of the similar features8:        **decrease** $K_{r}$9:    **end if**10:**end for**


#### 3.3.1. Clustering Strategy as a Scale-Up Option

The pruning algorithm, Algorithm 1, may not scale well to a large set of neurons or move quadratically in terms of the number of neurons in its computation. To bypass this issue, we consider a clustering-based procedure to perform the pruning in a group-based manner. In detail, we cluster features based on their similarity: features with high similarity should be together. Before that, we decide the number of clusters according to different choices of compression rates. When the clustering result is confirmed, we choose one feature to retain, which could be the one closest to each cluster centroid, while all the other neurons of the same cluster should be eliminated.

The mutual information is used to decide a metric for the clustering procedure. Given the pairwise distances, we utilize multidimensional scaling (MDS) [39] to find coordinates in a pre-specified dimensionality. In detail, given two features $Z_{k}$ and $Z_{\ell }$, we compute their mutual information $I(Z_{k};Z_{\ell })$, and a set of pairwise mutual information is transformed into pairwise distances. Then, Equation (1) is used to find coordinates given a pre-specified space of a certain dimensionality.

(1) $$d\left(Z_{k},Z_{\ell }\right)=A\exp\left(-I\left(Z_{k};Z_{\ell }\right)\right),$$

where *A* is a constant, and we prefer a distance between 0 and 1. In the formula, the larger the mutual information between $Z_{k}$ and $Z_{\ell }$, the smaller the value $d(Z_{k},Z_{\ell })$ is. In the MDS-projected space, two features $Z_{k}$ and $Z_{\ell }$ being close together implies that they share more mutual information $I(Z_{k};Z_{\ell })$. Moreover, two features with more mutual information $I\left(Z_{k};Z_{\ell }\right)\geq T_{r}$ may end up in the same cluster, and the pruning strategy in Algorithm 1 could suggest the removal of one of them. That is, we group features into a cluster if they have high mutual information. Once we obtain the grouping result, only one feature per cluster is used as the representative. In the end, we have features in different clusters if the features have less pairwise mutual information than a threshold $T_{r}$.

#### 3.3.2. Subsidiary Condition

This procedure may not produce a unique compression model, because the solution to MDS and the selection of representatives may not always be the same. We suggest some subsidiary condition to encourage a decent compression result by trying *M* random seeds and choosing the best one according to the following criteria.

The subsidiary condition aims to minimize the difference between the original and compressed models. We use the Kullback–Leibler (KL) divergence to measure the difference between the original and the compressed model via Equation (2), if focusing on the representation of both models. Given the original model, we aim to find a compressed model M that is closest to the original model in its representation distribution when measured according to the KL divergence.

(2) $$M_{\mathrm{comp}}^{*}=\underset{M}{\mathrm{argmin}}D_{\mathrm{KL}}(p\left(z_{o}\right)||p\left(z_{M}\right))\;,$$

where $D_{\mathrm{KL}}\left(p_{1}∥p_{2}\right)$ measures the KL divergence between two distributions $p_{1}$ and $p_{2}$; $z_{o}$ and $z_{M}$ denote the representation of the original and the compressed model M, respectively; $p(z_{o})$ and $p(z_{M})$ are the distributions of $z_{o}$ and $z_{M}$, respectively. A small $D_{\mathrm{KL}}$ indicates a closer relationship between $p(z_{M})$, the representation for the compressed model, and $p(z_{o})$, the representation of the original model.

### 3.4. Estimation Method of Mutual Information Between Hidden Neurons

The existing matrix-based Rényi α-order entropy is mainly used to estimate the mutual information (MI) between inputs and hidden neurons or between outputs and hidden neurons but is rarely applied to estimate the MI between hidden neurons themselves. Therefore, we propose a matrix-based Rényi α-order entropy estimation method specifically designed for hidden neurons.

#### 3.4.1. Matrix-Based Rényi α-Order Entropy

Given random variables $Z=\left\{z_{1},z_{2},\ldots ,z_{n}\right\}$ and the Gram matrix *K*, derived by evaluating a positive definite kernel *k* on every pair of data points such that ${\left(K\right)}_{ij}=k\left(x_{i},x_{j}\right)$, a matrix-based formulation of Rényi α-order entropy can be defined for a normalized positive definite matrix *A* (size n×n), where tr(A)=1, using the following functional expression:

(3) $$S_{\alpha }\left(A\right)=\frac{1}{1-\alpha }\log_{2}\left[\sum_{i=1}^{n}\lambda_{i}{\left(A\right)}^{\alpha }\right]\;.$$

#### 3.4.2. Matrix-Based Rényi α-Order Joint Entropy

Given *n* pairs of samples ${\left\{Z=(x_{i},y_{i})\right\}}_{i=1}^{n}$, each sample contains two different types of measurements x∈X and y∈Y and the positive definite kernels $K_{\infty }:X\times X\to R$ and $K_{\in }:Y\times Y\to R$; then, the matrix-based formulation of Rényi’s α-order joint entropy can be defined as follows:

(4) $$S_{\alpha }\left(A,B\right)=S_{\alpha }\left(\frac{A\circ B}{tr(A\circ B)}\right)\;,$$

where $A_{ij}=K_{\propto }\left(x_{i},x_{j}\right)$, $B_{ij}=K_{\in }\left(y_{i},y_{j}\right)$, and A∘B denotes the Hadamard product between the matrices *A* and *B*.

#### 3.4.3. MI Expressed Through Matrix-Based Rényi’s α-Order Entropy

(5) $$I_{\alpha }\left(A,B\right)=S_{\alpha }\left(A\right)+S_{\alpha }\left(B\right)-S_{\alpha }\left(A,B\right)\;,$$

where $S_{\alpha }\left(A\right)$ and $S_{\alpha }\left(B\right)$ denote the matrix-based Rényi’s α-order entropy in Equation (3), and $S_{\alpha }\left(A,B\right)$ denotes the matrix-based Rényi’s α-order joint entropy in Equation (4).

#### 3.4.4. Estimation Method of the Kernel Width Parameter of a Hidden Neuron

The key to the matrix-based Rényi α-order entropy estimator is the estimation of the kernel width parameter. Suppose that there are *n* hidden neurons, and their activation outputs are represented as $Z_{1},Z_{2},\ldots ,Z_{n}$. When we want to estimate the mutual information between different $Z_{i}$, we map the the random variables $Z_{1},Z_{2},\ldots ,Z_{n}$ to a reproducing kernel Hilbert space (RKHS) first, where the Gaussian kernel can be expressed as

(6) $$K_{\sigma }\left(Z_{i},Z_{j}\right)=\exp\left(-\frac{\left|\right|Z_{i}-Z_{j}{\left|\right|}_{F}^{2}}{2\sigma^{2}}\right)\;,$$

where $\left|\right|\cdot {\left|\right|}_{F}$ denotes the Frobenius norm.

The asymptotic behavior of entropy by varying σ can be denoted by

(7) $$lim_{\sigma \to 0}S_{\alpha }\left(A\right)=\log N,$$

(8) $$lim_{\sigma \to \infty }S_{\alpha }\left(A\right)=0.$$

High-dimensional and large-scale input features lead to a similar effect as a small σ, causing the entropy to be overestimated. Conversely, low-dimensional and small-scale input features have the same impact as a large σ, resulting in the entropy being underestimated [32]. Thus, appropriate hyperparameter tuning for σ is essential to prevent the excessive or insufficient saturation of entropy during training.

There are two existing estimation methods, one of which is Scott’s rule [38], considered for the unsupervised learning case. However, when estimating mutual information (MI) in deep neural networks (DNNs), the high-dimensional nature of the data often leads to the failure of unsupervised heuristic methods. An alternative is the optimal criterion [19], which follows a supervised approach. This method maximizes the kernel alignment loss between the kernel matrix of a given layer and the label kernel matrix. Although it outperforms Scott’s rule [38], it requires access to the label kernel matrix.

In our case, we want to estimate the MI between two hidden neurons in the high- dimensional DNN network, and we do not have the label kernel matrix. Therefore, we start by creating the label kernel matrix and then use the optimal criterion to estimate the kernel width parameter for every hidden neuron.

The process is illustrated as follows.

First, we utilize Scott’s rule to determine the kernel width parameter $\sigma^{\ell }$ for the target hidden layer, resulting in

(9) $$\sigma^{\ell }=\gamma N^{\frac{-1}{\left(4+d\right)}}\;,$$

where *N* denotes the number of samples, *d* denotes the the number of hidden neurons, and γ is an empirically determined constant. Second, we adopt Equation (6) to calculate the kernel matrix $K_{\sigma^{\ell }}$ of the hidden layer. The RBF kernel is written as Equation (6). Third, we align the kernel matrix $K_{\sigma^{\ell }}$ with the kernel matrix $K_{\sigma^{n}}$ (representing the hidden layer) by optimizing the kernel alignment loss between them. The kernel alignment loss [40] is expressed as

(10) $$A\left(K_{\sigma^{\ell }},K_{\sigma^{n}}\right)=\frac{{\langle K_{\sigma^{\ell }},K_{\sigma^{n}}\rangle }_{F}}{{∥K_{\sigma^{\ell }}∥}_{F}{∥K_{\sigma^{n}}∥}_{F}}\;.$$

Here, ${∥\cdot ∥}_{F}$ represents the Frobenius norm, and ${\langle \cdot ,\cdot \rangle }_{F}$ denotes the associated inner product.

Accordingly, the optimal $\sigma^{n}$ is selected, as shown in Equation (11):

(11) $$\sigma^{n*}=\underset{\sigma^{n}}{\arg\max}A\left(K_{\sigma^{l}},K_{\sigma^{n}}\right)\;.$$

The optimal value of $\sigma^{n}$ depends on the mini-batch size. To compute its final value, we employ the approach outlined by Wickström et al. [19], which uses an exponential moving average, as follows:

(12) $$\sigma_{n,t}=\beta \sigma_{n,t-1}+\left(1-\beta \right)\sigma_{n,t}^{*}\;,$$

where β∈[0,1], and $\sigma_{n,1}=\sigma_{n,1}^{*}$.

Finally, we determine the kernel width parameter for each hidden neuron within the hidden layer.

## 4. Results of the Experiments

We conducted a series of experiments to evaluate the effectiveness of the proposed model. The first goal was to understand the accuracy of the mutual information estimation of the hidden neurons. Then, we needed to confirm the effectiveness of the proposed pruning method. Relative FLOPs are used to indicate the compression level of the model, and the formula is expressed as

$$\mathrm{Relative}\;\mathrm{FLOPs}=\frac{\mathrm{FLOPs}\;\mathrm{of}\;\mathrm{pruned}\;\mathrm{model}}{\mathrm{FLOPs}\;\mathrm{of}\;\mathrm{original}\;\mathrm{model}}\;.$$

The smaller the value of the relative FLOPs, the higher the compression level of the model.

### 4.1. Experimental Settings

We evaluated the effectiveness of the proposed methods using the BERT-tiny model [41] on the General Language Understanding Evaluation (GLUE) [42] benchmark. The BERT-tiny is a (pretraining + fine-tuning) model from [41], which consists of one embedding layer and two Transformer encoder blocks, with a hidden size of 512 for the FC layer in FFN. The GLUE benchmark contains a collection of NLU tasks, and we fine-tuned it on five downstream tasks: Single-Sentence Task (SST-2 [43]), Similarity and Paraphrase Tasks (STS-B [44], MRPC [45], QQP [45]), and Inference Task (QNLI [45]). The batch sizes were set to 8, 8, 8, 16, and 16 for these tasks, respectively. Additionally, the learning rates were set to $5\times 10^{-4}$, $5\times 10^{-4}$, $3\times 10^{-4}$, $3\times 10^{-4}$, and $3\times 10^{-4}$ for these tasks, respectively. Throughout all experiments, we trained the model using the AdamW optimizer [46] with $\beta_{1}=0.9$, $\beta_{2}=0.999$, and $\epsilon =10^{-8}$, and conducted a total of four fine-tuning epochs. The overall data statistics and corresponding evaluation metrics are shown in Table 2.

To calculate the mutual information settings, we randomly sampled 1% of the number of samples per task training dataset, to determine that the *N* in Equation (9) is equal to the random sample number. At the same time, in Equation (5), we set α = 1.01, and, in Equation (9), we set γ = 1, *n* = 512. The batch size of the calculation process was set to 100, which was the same as in [31]. Due to the randomness of MDS, we sampled 500 random seeds and chose the best one based on Equation (2). For the larger architectures of BERT, such as ${\mathrm{BERT}}_{\mathrm{base}}$, we randomly sampled 10 samples from the training dataset.

### 4.2. The Results of Model Pruning

In this part, we compare the proposed method to three other types: some supervised learning approaches [26,27,28], a self-supervised learning method [27], and unsupervised learning methods [3,17]. The proposed method is similar to the weight-magnitude approach [3] and KCM [17], in the sense that none of these need labeled data in the pruning procedure. Moreover, the proposed method is a retraining-free approach, which follows the convention from [17,26,28]. It is different from the approach adopted in [17,26,28], where weight-tuning on the left-out (unpruned) neurons is necessary to confirm more-than-acceptable network effectiveness. Note that the random strategy has its output as an average of ten trials to reveal its general behavior.

**Figure 2:**
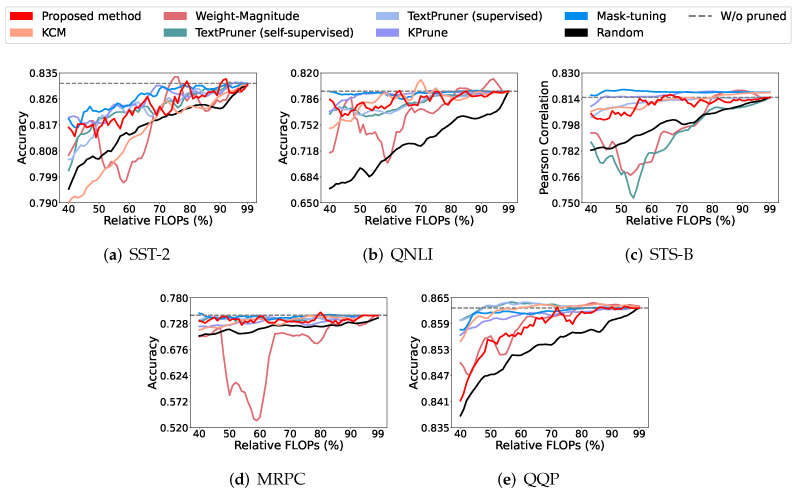
Performance of the proposed method against other methods on BERT-tiny. Testing was conducted using the dev set to control the relative FLOPs incrementally by percentage. The red line represents our proposed method, while the pink curve corresponds to the weight-magnitude method.

To clearly show the model performance under different compression rates, we consider the following set of comparisons, given five tasks. As shown in Figure 2, we demonstrate the result for every 1% change in the compression rate. For all the tasks other than STS-B, the proposed method performs better than the random strategy. Compared to the unsupervised learning method proposed by Li et al. [3], our proposed pruning method shows better performance on all except the QQP dataset, for which both perform similarly. In the unsupervised group, we also consider KCM [17]. In this case, both perform similarly on all four, except the QQP dataset. The proposed method shows slightly better performance on SST-2.

For the self-supervised method from Yang et al. [27], the proposed method shows its advantage on the QNLI and STS-B datasets but not on the SST-2 dataset. In the category of supervised learning, the proposed method shows similar results on both the STS-B and QQP datasets given low compression rates; however, it performs poorly given high compression rates. On the SST-2, QNLI, and MRPC datasets, all behave similarly.

In Table 3, we show that the proposed model can maintain the performance of the original model for various tasks when the relative FLOPs are set to 40%. Let us compare the proposed method to the weight-magnitude [3] and KCM [17] approaches, which are unsupervised methods and somewhat similar to our method. The proposed method has superior performance to the weight-magnitude method on the SST-2 and the QNLI, while it performs slightly worse on the other three tasks. Compared to the self-supervised method of Yang et al. [27], the proposed method is superior on all but the MRPC and QQP tasks. Finally, we evaluated the superiority of the proposed method to supervised methods. In this category, the proposed method performed better than the other two methods on the SST-2 and QNLI tasks. For instance, the proposed method was better than mask-tuning [26] on the SST-2 and QNLI tasks and better than Yang et al. [27] on the SST-2 task. For other tasks, the proposed method and the two supervised methods performed similarly.

Table 4 reports the SST-2 results. The proposed method maintains the original model’s performance while constraining the relative FLOPs to 40%, 50%, 60%, 70%, 80%, and 90%. Compared with the unsupervised KCM approach [17], our method outperforms KCM at 40% and 50% relative FLOPs and remains comparable at 80% and 90%. A similar pattern is shown with the self-supervised TextPruner of Yang et al. [27]: our method is better at 40% relative FLOPs and comparable at 80% and 90%. Relative to the supervised baselines, our method is likewise competitive, with comparable results at 40%, 80%, and 90% relative FLOPs.

### 4.3. Explanation of the Network Pruning

In this subsection, we employ the RPI (randomized path-integration) explainability method to provide visualized insights into the relationship between the input and the model’s predictions during the compression process. RPI introduces randomized baseline sampling and performs path integration on the attention scores and their gradients, effectively generating a set of candidate attribution maps. The most appropriate attribution map is then selected based on specific evaluation metrics, thereby enhancing the interpretability of language models [47].

Following the methodology of [47], our analysis applies RPI by integrating over the internal attention scores rather than the token embeddings themselves. The gradients are calculated with respect to the model’s final prediction (i.e., the sentiment class logit). As our base model is BERT, and aligning with the implementation in the RPI paper, this integration is performed on the final layer of the model, and the resulting attribution scores are extracted from the row corresponding to the [CLS] token. This process allows us to aggregate information from the final layer’s attention heads to determine each input token’s contribution to the classification task.

**Figure 3:**
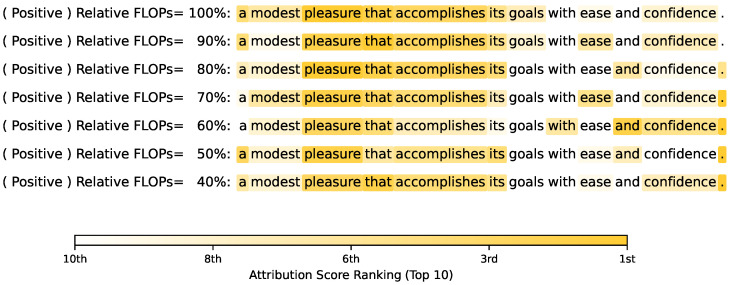
The RPI attribute score visualization for ${\mathrm{BERT}}_{\mathrm{base}}$ with different relative FLOPs pruned according to an incremental approach.

We probe the internal mechanisms of the pruned models by visualizing the RPI attribution scores for ${\mathrm{BERT}}_{\mathrm{base}}$ on the SST-2 dataset, with varying levels of relative FLOPs pruned using an incremental approach. As illustrated for a positive-sentiment instance in Figure 3, we track the attribution maps as our MI-based incremental method compresses the model from 100% down to 40% of its original relative FLOPs.

The visualization reveals a remarkably stable reasoning process. The unpruned model (100% FLOPs) correctly attributes its positive prediction to the key semantic drivers “modest,” “pleasure,” and “confidence.” Critically, even after aggressive pruning to 40% FLOPs, the model’s attribution map remains steadfastly focused on these same tokens.

This stability is non-trivial, as prior work [48] has shown that naive pruning methods can disrupt internal logic, yielding unstable or noisy attributions that rely on spurious correlations (e.g., focusing on “with” or “goals”). Therefore, the observed consistency provides strong evidence that our MI-based approach successfully identifies and removes computational redundancy—such as co-dependent neurons or attention heads—while meticulously preserving the core linguistic reasoning pathways vital to the task.

This analysis demonstrates that the pruned model does not merely maintain accuracy by chance. It retains a consistent and interpretable internal logic, confirming the precision and efficacy of our pruning strategy.

### 4.4. Mutual Information Between Hidden Neurons Estimation

In this part of experiment, we examined the MI estimation between hidden neurons. We compare the adopted approach and the estimation based on the kernel width parameter tuning (Scott’s rule). Of course, no true value can be obtained to confirm the accuracy of the MI estimation; however, we provide this study on the model test accuracy as further support for the pruning effectiveness.

**Figure 4:**
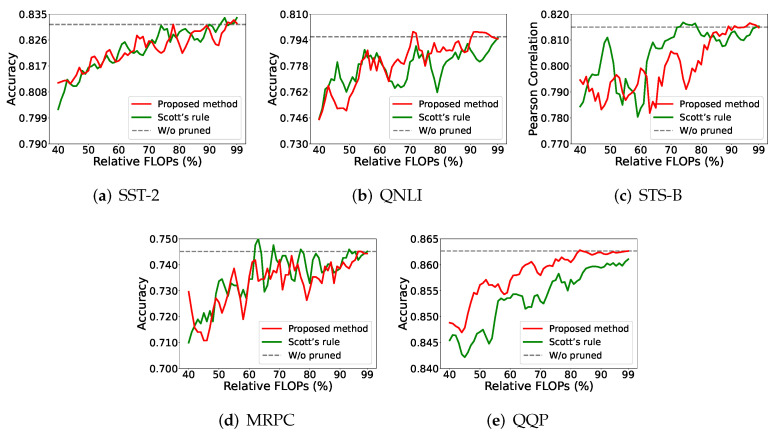
Performance of the proposed method against other MI estimators on BERT-tiny. Testing was conducted using the dev set to control the relative FLOPs incrementally by percentage.

As shown in Figure 4, the proposed method has better performance in terms of the prediction accuracy than other methods in most cases. It has a clear advantage on the QQP dataset, across all compression rates. On the QNLI dataset, the proposed method still retains an advantage in most cases. On the SST-2 and MRPC datasets, both (the proposed method and Scott’s rule) show similar results. Finally, the proposed method performs poorly on the STS-B dataset, for the low-compression-rate cases.

### 4.5. Ablation Study

We examined some alternative approaches to determine the possibility of further improving the proposed method.

#### 4.5.1. Mutual Information vs. Pearson Correlation Coefficient

**Figure 5:**
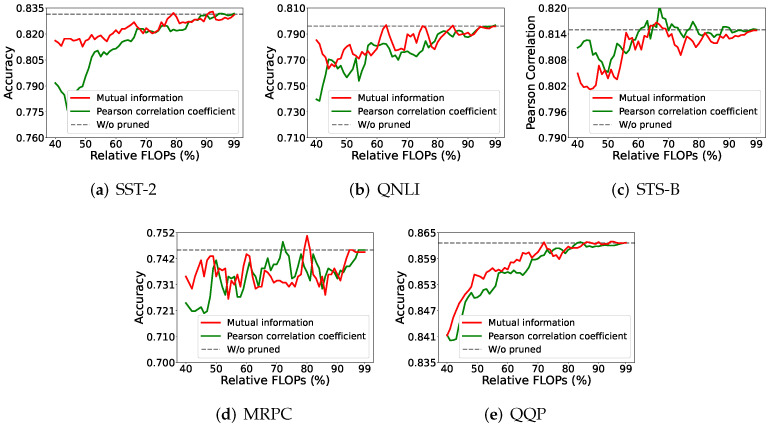
The results based on mutual information or Pearson correlation coefficient computation.

First, we considered an alternative measure to describe how two variables are related to each other, when we measure the relation between two groups of neurons. We chose to substitute the Pearson correlation coefficient (PCC) for MI in Algorithm 1 to guide the pruning procedure. The results are shown in Figure 5.

In Figure 5, using MI, rather than PCC, to describe the relation between neurons performs better in the high-compression (small remaining model) situations, for all but the STS-B dataset. This implies that MI can offer complex descriptions between different random variables, compared to the PCC, which covers only simple or linear relationships between random variables. That is to say, the pruning on a large model may not need careful treatment because neurons may find their substitutes easily, while the pruning on a small model needs precise calculations, which can be achieved using the MI-based approach.

#### 4.5.2. Data Samples for Mutual Information Estimation

This series of experiments was devoted to studying the quantity of data needed to estimate the MI value. If a small dataset offers a result similar to a large dataset, then we would use a small dataset to save the run time in model pruning.

When estimating the MI value, we rely on the values of the hidden neurons. That is, once the network is built after a converged training, we can sample a small portion of the input data and use them to activate the hidden neurons for the estimation. According to our study, the estimation does not need large-scale data to confirm the pruning effect.

**Figure 6:**
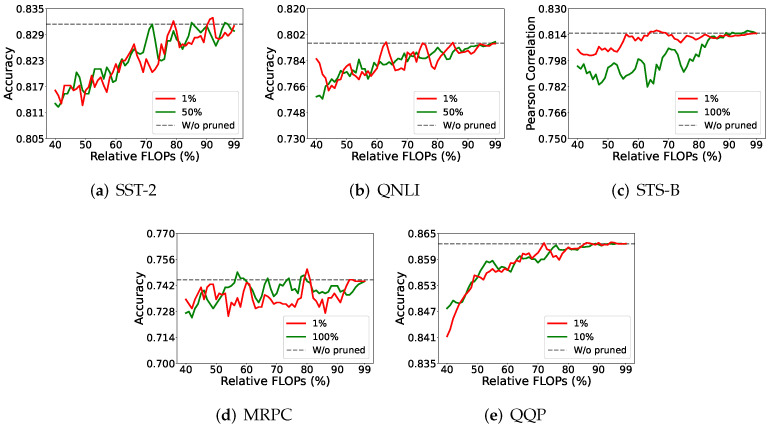
Insignificant difference between the result from either the complete or partial dataset.

As shown in Figure 6, we considered 1%, 10%, 50%, or 100% (all) of the complete data to see their pruning performance. Different data portions still provide similar model performance on all but the STS-B dataset. Note that we need a regression task for the STS-B dataset, while classification tasks are needed for all other datasets.

#### 4.5.3. Different α for Matrix-Based Rényi α-Order Entropy Estimation

**Figure 7:**
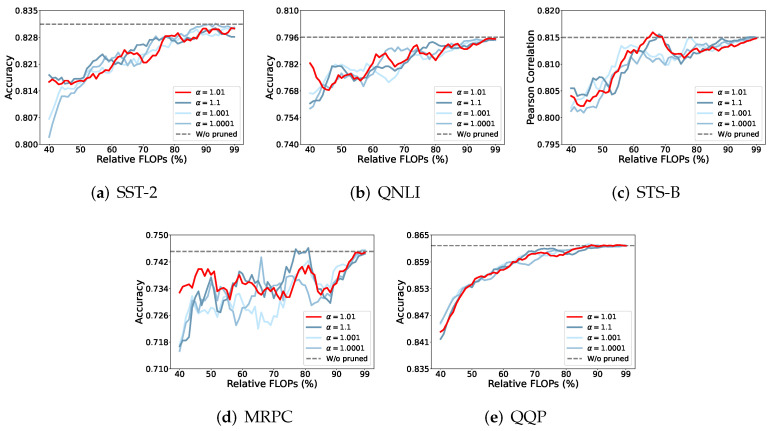
The value of α=1.01 demonstrates exceptional performance in highly pruned models compared to other α values utilized in Rényi entropy estimation.

In this series of experiments, we aimed to determine the best choice of α, for the estimation of Rényi α-order entropy. As shown in Figure 7, choosing α=1.01 provided the best result for the five different tasks.

#### 4.5.4. Sample Number for MDS

**Figure 8:**
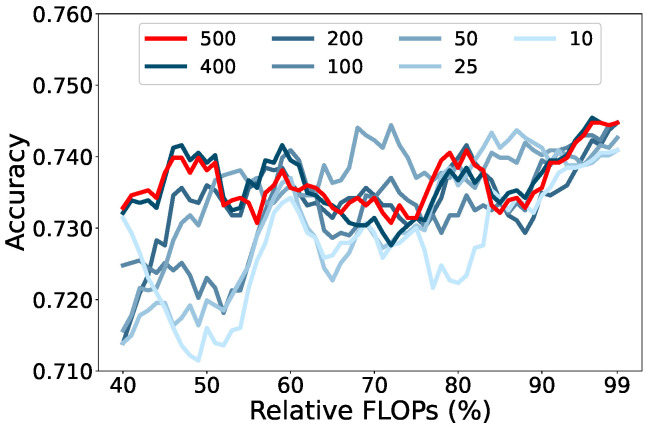
In the MRPC experiment, the performance converges when the number of samples exceeds 400.

The proposed procedure may not always produce the same unique compressed model because the solution to MDS and the selection of representatives may not remain the same. To deal with this, we sampled a few random seeds and chose the best one based on Equation (2), and the result is shown in Figure 8.

## 5. Limitations

The current methodology demonstrates limited robustness when applied to datasets characterized by a very large scale and inherent noise or complex linguistic patterns, notably the Quora Question Pairs (QQP) dataset. At high compression ratios (e.g., relative FLOPs >50%), the challenge lies in effectively retaining the necessary fine-grained semantic dependencies. In this specific context, the method struggles to fully preserve all critical information captured within the deep hidden representations, leading to a noticeable context-specific performance degradation. Future work will focus on enhancing the information-theoretic criteria to improve its stability and noise resilience in such demanding real-world datasets.

## 6. Conclusions

In this work, we propose a new MI-based structured pruning approach for BERT. We show that the method achieves high compression with minimal accuracy loss, a result backed by an empirically favorable trade-off rooted in the minimization of the KL divergence between model representations, making it suitable for on-device deployment. Our approach is unsupervised, requires no labeled data, and utilizes a tailored MI estimation technique with a novel kernel bandwidth estimator for better accuracy. We also introduced visualizations to enhance the interpretability of the compression process, showing changes in model representations and predictions. Based on a series of experiments, we conclude that the proposed method produced a pruned model that is both more effective and significantly smaller than those resulting from other state-of-the-art pruning approaches. Its superiority includes almost all unsupervised approaches and a few supervised approaches. The result is also similar to that offered by self-supervised learning. In the future, we would like to take an even larger model than what we can process now to confirm the scalability of the proposed method.

## Figures and Tables

**Table 1:** Summary of MI estimation methods.

Method	Core Principle	Primary Advantage	Key Limitation
Binning-based [30]	Quantizes neuron output to estimate probability distributions.	Conceptually straightforward and easy to implement.	Sensitive to bin size; requires large sample sizes; struggles with high-dimensional non-linearities.
KSG [34]	Estimates MI based on *k*-nearest neighbor distances in high-dimensional space.	Applicable to a wide range of activation functions and density forms.	Highly sensitive to the choice of the number of neighbors (*k*).
MINE [35]	Uses an auxiliary neural network trained via gradient descent to estimate MI.	Scales linearly with dimensionality and sample size; excellent for high-dimensional data.	Slow convergence speed; highly sensitive to the auxiliary network’s architecture.
Rényi α-Order [19]	Estimates MI via the kernel width parameter (σ), avoiding direct probability estimation.	Does not require explicit probability density estimation; computationally efficient.	Critical reliance on σ estimation; existing methods often focus on the *entire* layer, not individual neurons.

**Table 2:** Data statistics of GLUE datasets. Among them, STS-B is a regression task, and the others are classification tasks.

Tasks	Datasets	Training	Validation	Test	Metrics
Single-sentence	SST-2	67,350	873	1821	Accuracy
Inference	QNLI	104,743	5463	5461	Accuracy
Similarity and paraphrase	STS-B	5749	1379	1377	Pearson correlation (*r*) Spearmen correlation ($r_{s}$)
	MRPC	3668	408	1725	F1/Accuracy
	QQP	363,870	40,431	390,965	F1/Accuracy

**Table 3:** Results of different methods when the relative FLOPs are equal to 40%. Abbreviations: S, Self-S, and U denote the supervised method, the self-supervised method, and the unsupervised method, respectively.

Methods	S/U /Self-S	Relative FLOPs	SST-2 Acc	STS-B *r*/$r_{s}$	MRPC Acc/F1	QQP Acc/F1	QNLI Acc
BERT-tiny (Original)		100%	83.2	74.3/73.6	81.1/71.1	62.2/83.4	81.5
TextPruner [27]	S	40%	80.8	72.9/70.5	**81.3/70.7**	62.7/85.3	**78.7**
Mask-tuning [26]	S	40%	81.7	73.7/70.9	80.7/69.6	61.8/85.3	65.0
Kprune [28]	S	40%	**83.1**	**74.4/72.3**	81.0/70.1	61.8/84.2	77.5
TextPruner [27]	Self-S	40%	81.8	70.3/68.7	80.8/70.0	**62.8/84.9**	76.2
Random	U	40%	80.7	71.0/69.4	80.8/68.7	59.1/84.4	67.2
Weight-Magnitude [3]	U	40%	81.8	71.4/69.6	80.8/68.5	61.2/83.9	67.7
KCM [17]	U	40%	78.8	72.6/70.3	81.1/69.8	61.9/84.0	74.5
**Proposed method**	U	40%	82.6	72.1/69.2	80.9/69.4	61.2/84.3	77.2

TextPruner [27] includes two methods: one is self-supervised learning, and the other is supervised learning. Also, bold indicates the best result, and underlined indicates the second-best result.

**Table 4:** Comparison of run time and accuracy at different pruning ratios on the SST-2 tasks.

		Accuracy Under Specific Relative FLOPs					
**Method**	**S/U/Self-S**	**40%**	**50%**	**60%**	**70%**	**80%**	**90%**
${\mathrm{BERT}}_{\mathrm{base}}$ (original)		93.57					
TextPruner	S	63.99	83.60	87.04	88.30	92.20	**92.88**
MaskTuning	S	71.44	84.28	89.10	91.51	91.97	92.77
KPrune	S	50.33	49.08	49.54	50.57	51.61	51.49
KPrune *	S	**88.30**	**89.68**	**90.83**	**92.78**	**92.55**	92.78
TextPruner	SSL	62.84	84.17	84.97	90.36	92.08	92.43
KCM	U	52.86	74.19	83.48	88.07	91.85	91.97
**Proposed method**	U	65.25	76.14	75.34	84.97	91.05	91.85

KPrune * is processed with 235 instances; Also, bold indicates the best result, and underlined indicates the second-best result.

## Data Availability

No new data were created or analyzed in this study. Data sharing is not applicable to this article.
